# Supplementary material for: The Cross Marks the Spot: The Emerging Role of JmjC Domain-Containing Proteins in Myeloid Malignancies
Source: Biomolecules. 2021 Dec 20;11(12):1911. doi: 10.3390/biom11121911 (PMC8699298; doi:10.3390/biom11121911)
Supplement: Supplementary file 1 [file biomolecules-11-01911-s001.zip › biomolecules-1498771-supplementary.pdf]

Table S1: Abbreviations of relevant proteins

Abbreviation	Full name	Classification	Comment	References
ALKBH5	alpha-ketoglutarate-dependent dioxygenase alkB homolog 5	RNA N^6-methyladenosine demethylase	ALKBH5 is involved in various biological processes including proliferation and plays an emerging role in carcinogenesis.	[1–3]
ASH1L	absent small and homeotic disks protein 1 homolog	histone-lysine N-methyltransferase	ASH1L catalyzes H3K36 methylation, is involved in *HOX* gene regulation and cooperates with MLL.	[4–8]
AXL	AXL Receptor Tyrosine Kinase	Tyro3-Axl-Mer (TAM) receptor tyrosine kinase	AXL stimulates PI3K/AKT/mTOR signaling and is constitutively active in AML.	[2,9–11]
BCL2	B-cell lymphoma 2 protein	Bcl2 protein / apoptosis regulator protein	BCL2 is an anti-apoptotic protein.	[12,13]
BCL2L11 (BIM)	Bcl-2-like protein 11	Bcl2 protein / apoptosis regulator protein	BCL2L11 (BIM) is a pro-apoptotic protein.	[12–14]
BRG1 (SMARCA4)	SWI/SNF-related matrix-associated actin-dependent regulator of chromatin subfamily A member 4	SWI/SNF (SWItch/Sucrose Non-Fermentable) family member	BRG1 (SMARCA4) is a member of the BAF family of ATP-dependent chromatin-remodeling complexes and mentioned as PRC2 antagonist.	[15,16]
CCND2	cyclin D2	cyclin protein	CCND2 is involved in cell cycle G1/S transition.	[17]
CDKN1A (p21)	cyclin-dependent kinase inhibitor 1	cyclin-dependent kinase inhibitor protein	CDKN1A (p21) is a p53 target and mediates cell cycle arrest.	[18–20]
CDX2	homeobox protein CDX-2	homeodomain-containing transcription factor	CDX2 is involved in AML transformation.	[21,22]
CEBPA	CCAAT/enhancer-binding protein alpha	basic leucine zipper (bZIP) transcription factor	CEBPA is essential for myeloid lineage commitment.	[23]
CEBPB	CCAAT/enhancer-binding protein beta	basic leucine zipper (bZIP) transcription factor	CEBPB is essential for macrophage differentiation and involved in immune as well as in inflammatory responses.	[24–26]
DOCK5/8	dedicator of cytokinesis 5/8 proteins	guanine nucleotide exchange factors	DOCK5 and DOCK8 are involved in cancer cell survival through Ras-mediated activation of the Rac pathway.	[27, 28]
EGR1	early growth response protein 1	zinc finger (ZF) transcription factor	EGR1 acts context dependent as tumor suppressor or oncogene and is widely seen as tumor suppressor in AML.	[29]
ENT1	equilibrative nucleoside transporter 1	drug influx transporter	ENT1 is involved in the cellular uptake of nucleosides and its analogues.	[30]
EZH2	enhancer of zeste homolog 2	histone-lysine N-methyltransferase enzyme	EZH2 is a tumor suppressor in AML/MDS.	[31–33]
GATA1	GATA-binding factor 1	GATA transcription factor	GATA1 is essential for erythroid and megakaryocytic differentiation.	[34–36]
HOXA9	homeobox protein Hox-A9	homeodomain-containing transcription factor	HOXA9 is commonly dysregulated in acute leukemias and involved in leukemic transformation. It interacts with MEIS1.	[37,38]
HOXA10	homeobox protein Hox-A10	homeodomain-containing transcription factor	HOXA10 is mentioned as PRC2 antagonist.	[15]
HP1	heterochromatin protein 1	heterochromatin protein 1 family	HP1 proteins are involved in heterochromatin formation.	[39–41]
ID1	DNA-binding protein inhibitor ID-1	helix-loop-helix (HLH) protein	ID1 interacts with and inhibits HLH transcription factors.	[42]
IL3RA	Interleukin 3 receptor, alpha	interleukin receptor	IL3RA is a subunit of the heterodimeric IL3 receptor.	[43,44]

IL6	interleukin 6	interleukin	IL6 is a proinflammatory cytokine. It is involved in proliferation and differentiation of hematopoietic stem and progenitor cells (HSPCs).	[45–48]
IRF	interferon regulatory factors	interferon regulatory transcription factor (IRF) family	IRF proteins are essential for transcriptional regulation of type I IFN genes.	[49,50]
JAK2	janus kinase 2	non-receptor tyrosine kinase	$JAK2^{V617F}$ is the most frequent mutation in MPN patients.	[51]
JUN-AP1	activator protein 1	heterodimeric basic leucine zipper (bZIP) transcription factor	The oncoprotein JUN-AP is highly expressed in AML patients and involved in AML cell growth as well as proliferation.	[52–55]
KRAS	Kirsten rat sarcoma virus protein	GTPase	The oncoprotein KRAS mediates signal transduction of growth factors. Gain-of-function mutations occur in various cancers including leukemias.	[56–59]
LAPTM4B	lysosomal-associated transmembrane protein 4B	lysosome-associated protein transmembrane (LAPTM) family	LAPTM4B is required for lysosomal organization and involved in chemotherapy resistance. It predicts poor prognosis in AML.	[60–62]
LEDGF	lens Epithelium Derived Growth Factor	epigenetic reader protein	LEDGF associates with MLL-fusions and is required for MLL-rearranged leukemogenesis.	[4,63–67]
LEO1	RNA polymerase-associated protein LEO1	member of the polymerase-associated factor 1 (PAF1) complex	As member of the PAF1 complex LEO1 is involved in leukemogenesis through association with MLL-fusions.	[68–70]
LMO2	LIM domain only 2 protein	LIM-domain-only (LMO) protein	LMO2 regulates transcription as scaffolding protein and is involved in erythroid differentiation trough complex building with GATA1.	[71–73]
LSD1	lysine-specific histone demethylase 1A	histone demethylase	LSD1 demethylates $H3K4me^{1+2}$ and $H3K9me^{1+2}$ and is often deregulated in AML.	[74–76]
MCL1	myeloid cell leukemia-1 protein	Bcl2 protein / apoptosis regulator protein	MCL1 is an anti-apoptotic protein.	[13,77]
MEIS1	homeobox protein Meis1	homeodomain-containing transcription factor	MEIS1 is involved in megakaryocytic lineage development and often dysregulated in acute leukemias. It interacts with HOXA9.	[37,78,79]
MLL1	mixed-lineage leukemia 1 protein	histone-lysine N-methyltransferase	MLL1 methylates H3K4 and is involved in Hox gene regulation. Oncogenic fusions with other proteins often occur in acute leukemias.	[80–82]
MOZ	monocytic leukemia zinc finger protein	histone acetyltransferase (HAT)	MOZ regulates RUNX1 expression and contributes to leukemogenesis as fusion with TIF2.	[83,84]
MYC	myelocytomatosis protein	basic helix–loop–helix (bHLH) transcription factor	MYC promotes cell growth and proliferation. It is a known proto-oncogene in many human cancers.	[85]
NFE2	nuclear factor, erythroid-derived 2	heterodimeric basic leucine zipper (bZIP) transcription factor	NFE2 is involved in megakaryocytic lineage development and deregulated in most MPN patients.	[86,87]
MEIS1	homeobox protein Meis1	homeodomain-containing transcription factor	MEIS1 is involved in megakaryocytic lineage development and often dysregulated in acute leukemias. It interacts with HOXA9.	[88–92]
NGF	nerve growth factor	neurotrophin growth factor	NGF mediates survival, growth and proliferation through TRKA binding.	[93,94]
NOM1	nucleolar protein with MIF4G domain 1	MIF4G/MA3 domain-containing protein	NOM1 interacts with translation initiating proteins.	[95,96]

NSG2	neuron-specific gene 2 protein	neuron-specific gene family of small, single-pass transmembrane proteins	NSG2 is involved in vesicle trafficking and neural differentiation. It has been shown to impair HSC differentiation.	[97–100]
NUP98	nuclear pore complex protein Nup98-Nup96	nuclear pore complex protein	NUP98 contributes to nuclear-cytoplasmic trafficking and is often rearranged in acute leukemias.	[101–103]
p15(Ink4b)	cyclin-dependent kinase 4 inhibitor B	cyclin-dependent kinase inhibitor protein	p15(Ink4b) acts as a tumor suppressor.	[104–106]
PAF1	RNA polymerase II-associated factor 1 homolog	component of the polymerase-associated factor 1 (PAF1) complex	PAF1 is essential for transcriptional elongation.	[69,107,108]
PML	promyelocytic leukemia protein (PML)	tripartite motif (TRIM) protein family	The PML/RARα fusion mediates the differentiation block in most APL patients.	[109–112]
PRL-3	protein of regenerating liver-3	prenylated protein tyrosine phosphatase	The physiological role of PRL-3 remains unclear. It is involved in metastasis and frequently overexpressed in AML patients.	[113,114]
RARα	retinoic acid receptor alpha	nuclear receptor	RARα mediates retinoid signaling. The PML/RARα fusion mediates the differentiation block in most APL patients.	[111,115]
RUNX1	runt-related transcription factor 1	runt-domain transcription factor	RUNX1 is required for HSPC homeostasis and expansion. The RUNX1/RUNX1T1 fusion often occurs in AML patients.	[116,117]
RUNX1T1	RUNX1 Partner Transcriptional Co-Repressor 1	zinc finger (ZF) transcription factor	RUNX1T1 mediates transcriptional repression. The RUNX1/RUNX1T1 fusion often occurs in AML patients.	[118–121]
S100A8/9	S100 calcium-binding protein A8/9	S100 protein family	S100A8 and S100A9 form the heterodimer calprotectin that is part of the innate immune system. Calprotectin is highly expressed in granulocytes, involved in inflammation and critical for blocking differentiation in leukemic cells.	[122–125]
SCL	stem cell leukemia protein	basic helix–loop–helix (bHLH) transcription factor	SCL is required for HSC development and also involved in terminal maturation of selected blood cells. It is often dysregulated in leukemias.	[126–128]
SPI1/PU.1	SFFV proviral integration site-1 (Spi-1) / purine rich Box-1 protein	erythroblast transformation specific (ETS) domain transcription factor	SPI1/PU.1 is involved in myeloid and B-cell differentiation. It is a known target of RUNX1 target. SPI1/PU.1 suppression is critical in leukemic transformation.	[129–131]
STAT1/2	signal transducer and activator of transcription 1/2	STAT transcription factors	STAT1/2 is involved in cytokine and growth factor signaling. It is dysregulated in various malignancies including cancer.	[132,133]
TAF1B	TATA box-binding protein-associated factor RNA polymerase I subunit B	TATA-binding protein (TBP) associated factor (TAFs)	TAF1B is required for initiation of transcription by RNA polymerase I.	[134–136]
TIF2	transcriptional mediators/intermediary factor 2	member of the p160 nuclear receptor transcriptional coactivator family (NRCoAs)	TIF2 contributes to leukemogenesis as fusion with MOZ.	[83]
TRKA	tropomyosin receptor kinase A	neurotrophic tyrosine kinase receptor	TRKA is a high affinity receptor for NGF and activates Ras/MAPK and PI3K/Akt signaling.	[93,137]

References

[1] G. Zheng *u. a.*, „ALKBH5 is a mammalian RNA demethylase that impacts RNA metabolism and mouse fertility“, *Mol Cell*, Bd. 49, Nr. 1, S. 18–29, Jan. 2013, doi: 10.1016/j.molcel.2012.10.015.

[2] J. Wang *u. a.*, „Leukemogenic Chromatin Alterations Promote AML Leukemia Stem Cells via a KDM4C-ALKBH5-AXL Signaling Axis“, *Cell Stem Cell*, Bd. 27, Nr. 1, S. 81-97.e8, Juli 2020, doi: 10.1016/j.stem.2020.04.001.

[3] J. Wang *u. a.*, „The biological function of m6A demethylase ALKBH5 and its role in human disease“, *Cancer Cell Int*, Bd. 20, S. 347, 2020, doi: 10.1186/s12935-020-01450-1.

[4] L. Zhu *u. a.*, „ASH1L Links Histone H3 Lysine 36 Dimethylation to MLL Leukemia“, *Cancer Discov*, Bd. 6, Nr. 7, S. 770–783, Juli 2016, doi: 10.1158/2159-8290.CD-16-0058.

[5] K. A. McMahon *u. a.*, „Mll has a critical role in fetal and adult hematopoietic stem cell self-renewal“, *Cell Stem Cell*, Bd. 1, Nr. 3, S. 338–345, Sep. 2007, doi: 10.1016/j.stem.2007.07.002.

[6] C. D. Jude, L. Climer, D. Xu, E. Artinger, J. K. Fisher, und P. Ernst, „Unique and independent roles for MLL in adult hematopoietic stem cells and progenitors“, *Cell Stem Cell*, Bd. 1, Nr. 3, S. 324–337, Sep. 2007, doi: 10.1016/j.stem.2007.05.019.

[7] M. Jones *u. a.*, „Ash1l controls quiescence and self-renewal potential in hematopoietic stem cells“, *J Clin Invest*, Bd. 125, Nr. 5, S. 2007–2020, Mai 2015, doi: 10.1172/JCI78124.

[8] J. L. Hess, B. D. Yu, B. Li, R. Hanson, und S. J. Korsmeyer, „Defects in yolk sac hematopoiesis in Mll-null embryos“, *Blood*, Bd. 90, Nr. 5, S. 1799–1806, Sep. 1997.

[9] I.-K. Park *u. a.*, „Receptor tyrosine kinase Axl is required for resistance of leukemic cells to FLT3-targeted therapy in acute myeloid leukemia“, *Leukemia*, Bd. 29, Nr. 12, S. 2382–2389, Dez. 2015, doi: 10.1038/leu.2015.147.

[10] I.-K. Park, A. Mishra, J. Chandler, S. P. Whitman, G. Marcucci, und M. A. Caligiuri, „Inhibition of the receptor tyrosine kinase Axl impedes activation of the FLT3 internal tandem duplication in human acute myeloid leukemia: implications for Axl as a potential therapeutic target“, *Blood*, Bd. 121, Nr. 11, S. 2064–2073, März 2013, doi: 10.1182/blood-2012-07-444018.

[11] C. Rochlitz *u. a.*, „Axl expression is associated with adverse prognosis and with expression of Bcl-2 and CD34 in de novo acute myeloid leukemia (AML): results from a multicenter trial of the Swiss Group for Clinical Cancer Research (SAKK)“, *Leukemia*, Bd. 13, Nr. 9, S. 1352–1358, Sep. 1999, doi: 10.1038/sj.leu.2401484.

[12] J. M. Hardwick und L. Soane, „Multiple functions of BCL-2 family proteins“, *Cold Spring Harb Perspect Biol*, Bd. 5, Nr. 2, S. a008722, Feb. 2013, doi: 10.1101/cshperspect.a008722.

[13] R. J. Youle und A. Strasser, „The BCL-2 protein family: opposing activities that mediate cell death“, *Nat Rev Mol Cell Biol*, Bd. 9, Nr. 1, S. 47–59, Jan. 2008, doi: 10.1038/nrm2308.

[14] M. N. Koenig *u. a.*, „Pro-apoptotic BIM is an essential initiator of physiological endothelial cell death independent of regulation by FOXO3“, *Cell Death Differ*, Bd. 21, Nr. 11, S. 1687–1695, Nov. 2014, doi: 10.1038/cdd.2014.90.

[15] J. Andricovich, Y. Kai, W. Peng, A. Foudi, und A. Tzatsos, „Histone demethylase KDM2B regulates lineage commitment in normal and malignant hematopoiesis“, *Journal of Clinical Investigation*, Bd. 126, Nr. 3, S. 905–920, Jan. 2016, doi: 10.1172/JCI84014.

[16] C. Kadoch und G. R. Crabtree, „Mammalian SWI/SNF chromatin remodeling complexes and cancer: Mechanistic insights gained from human genomics“, *Sci Adv*, Bd. 1, Nr. 5, S. e1500447, Juni 2015, doi: 10.1126/sciadv.1500447.

[17] M. Malumbres, „Cyclin-dependent kinases“, *Genome Biol*, Bd. 15, Nr. 6, S. 122, 2014, doi: 10.1186/gb4184.

[18] F. Bunz *u. a.*, „Requirement for p53 and p21 to sustain G2 arrest after DNA damage“, *Science*, Bd. 282, Nr. 5393, S. 1497–1501, Nov. 1998, doi: 10.1126/science.282.5393.1497.

[19] W. S. el-Deiry *u. a.*, „WAF1, a potential mediator of p53 tumor suppression“, *Cell*, Bd. 75, Nr. 4, S. 817–825, Nov. 1993, doi: 10.1016/0092-8674(93)90500-p.

[20] J. LaBaer *u. a.*, „New functional activities for the p21 family of CDK inhibitors“, *Genes Dev*, Bd. 11, Nr. 7, S. 847–862, Apr. 1997, doi: 10.1101/gad.11.7.847.

[21] V. P. S. Rawat *u. a.*, „Ectopic expression of the homeobox gene Cdx2 is the transforming event in a mouse model of t(12;13)(p13;q12) acute myeloid leukemia“, *Proc Natl Acad Sci U S A*, Bd. 101, Nr. 3, S. 817–822, Jan. 2004, doi: 10.1073/pnas.0305555101.

[22] C. Scholl *u. a.*, „The homeobox gene CDX2 is aberrantly expressed in most cases of acute myeloid leukemia and promotes leukemogenesis“, *J Clin Invest*, Bd. 117, Nr. 4, S. 1037–1048, Apr. 2007, doi: 10.1172/JCI30182.

[23] E. Ohlsson, M. B. Schuster, M. Hasemann, und B. T. Porse, „The multifaceted functions of C/EBPα in normal and malignant haematopoiesis“, *Leukemia*, Bd. 30, Nr. 4, S. 767–775, Apr. 2016, doi: 10.1038/leu.2015.324.

[24] S. Kinoshita, S. Akira, und T. Kishimoto, „A member of the C/EBP family, NF-IL6 beta, forms a heterodimer and transcriptionally synergizes with NF-IL6“, *Proc Natl Acad Sci U S A*, Bd. 89, Nr. 4, S. 1473–1476, Feb. 1992, doi: 10.1073/pnas.89.4.1473.

[25] S. K. Roy *u. a.*, „MEKK1 plays a critical role in activating the transcription factor C/EBP-beta-dependent gene expression in response to IFN-gamma“, *Proc Natl Acad Sci U S A*, Bd. 99, Nr. 12, S. 7945–7950, Juni 2002, doi: 10.1073/pnas.122075799.

[26] D. Ruffell *u. a.*, „A CREB-C/EBPbeta cascade induces M2 macrophage-specific gene expression and promotes muscle injury repair“, *Proc Natl Acad Sci U S A*, Bd. 106, Nr. 41, S. 17475–17480, Okt. 2009, doi: 10.1073/pnas.0908641106.

[27] M. Biswas, S. S. Chatterjee, L. D. Boila, S. Chakraborty, D. Banerjee, und A. Sengupta, „MBD3/NuRD loss participates with KDM6A program to promote DOCK5/8 expression and Rac GTPase activation in human acute myeloid leukemia“, *FASEB J*, Bd. 33, Nr. 4, S. 5268–5286, Apr. 2019, doi: 10.1096/fj.201801035R.

[28] H. Tajiri *u. a.*, „Targeting Ras-Driven Cancer Cell Survival and Invasion through Selective Inhibition of DOCK1“, *Cell Reports*, Bd. 19, Nr. 5, S. 969–980, Mai 2017, doi: 10.1016/j.celrep.2017.04.016.

[29] J. Tian, Z. Li, Y. Han, T. Jiang, X. Song, und G. Jiang, „The progress of early growth response factor 1 and leukemia“, *Intractable Rare Dis Res*, Bd. 5, Nr. 2, S. 76–82, Mai 2016, doi: 10.5582/irdr.2015.01049.

[30] M. Pastor-Anglada und S. Pérez-Torras, „Nucleoside transporter proteins as biomarkers of drug responsiveness and drug targets“, *Front Pharmacol*, Bd. 6, S. 13, 2015, doi: 10.3389/fphar.2015.00013.

[31] Y. Nagata und J. P. Maciejewski, „The functional mechanisms of mutations in myelodysplastic syndrome“, *Leukemia*, Bd. 33, Nr. 12, S. 2779–2794, Dez. 2019, doi: 10.1038/s41375-019-0617-3.

[32] G. Sashida *u. a.*, „Ezh2 loss promotes development of myelodysplastic syndrome but attenuates its predisposition to leukaemic transformation“, *Nat Commun*, Bd. 5, S. 4177, Juni 2014, doi: 10.1038/ncomms5177.

[33] J. Stomper *u. a.*, „Integrative study of EZH2 mutational status, copy number, protein expression and H3K27 trimethylation in AML/MDS patients“, *Clin Epigenetics*, Bd. 13, Nr. 1, S. 77, Apr. 2021, doi: 10.1186/s13148-021-01052-2.

[34] A. Caiulo *u. a.*, „Mapping the gene encoding the human erythroid transcriptional factor NFE1-GF1 to Xp11.23“, *Hum Genet*, Bd. 86, Nr. 4, S. 388–390, Feb. 1991, doi: 10.1007/BF00201840.

[35] K. R. Katsumura, A. W. DeVilbiss, N. J. Pope, K. D. Johnson, und E. H. Bresnick, „Transcriptional mechanisms underlying hemoglobin synthesis“, *Cold Spring Harb Perspect Med*, Bd. 3, Nr. 9, S. a015412, Sep. 2013, doi: 10.1101/cshperspect.a015412.

[36] S. Mizuta *u. a.*, „CCAR1/CoCoA pair-mediated recruitment of the Mediator defines a novel pathway for GATA1 function“, *Genes Cells*, Bd. 19, Nr. 1, S. 28–51, Jan. 2014, doi: 10.1111/gtc.12104.

[37] W. F. Shen, S. Rozenfeld, A. Kwong, L. G. Köm ves, H. J. Lawrence, und C. Largman, „HOXA9 forms triple complexes with PBX2 and MEIS1 in myeloid cells“, *Mol Cell Biol*, Bd. 19, Nr. 4, S. 3051–3061, Apr. 1999, doi: 10.1128/MCB.19.4.3051.

[38] C. T. Collins und J. L. Hess, „Role of HOXA9 in leukemia: dysregulation, cofactors and essential targets“, *Oncogene*, Bd. 35, Nr. 9, S. 1090–1098, März 2016, doi: 10.1038/onc.2015.174.

[39] J. C. Eissenberg, T. C. James, D. M. Foster-Hartnett, T. Hartnett, V. Ngan, und S. C. Elgin, „Mutation in a heterochromatin-specific chromosomal protein is associated with suppression of position-effect variegation in Drosophila melanogaster“, *Proc Natl Acad Sci U S A*, Bd. 87, Nr. 24, S. 9923–9927, Dez. 1990, doi: 10.1073/pnas.87.24.9923.

[40] T. C. James und S. C. Elgin, „Identification of a nonhistone chromosomal protein associated with heterochromatin in Drosophila melanogaster and its gene“, *Mol Cell Biol*, Bd. 6, Nr. 11, S. 3862–3872, Nov. 1986, doi: 10.1128/mcb.6.11.3862-3872.1986.

[41] A. Kumar und H. Kono, „Heterochromatin protein 1 (HP1): interactions with itself and chromatin components“, *Biophys Rev*, Bd. 12, Nr. 2, S. 387–400, Apr. 2020, doi: 10.1007/s12551-020-00663-y.

[42] R. Benezra, R. L. Davis, D. Lockshon, D. L. Turner, und H. Weintraub, „The protein Id: a negative regulator of helix-loop-helix DNA binding proteins“, *Cell*, Bd. 61, Nr. 1, S. 49–59, Apr. 1990, doi: 10.1016/0092-8674(90)90214-y.

[43] F. C. Stomski *u. a.*, „Human interleukin-3 (IL-3) induces disulfide-linked IL-3 receptor alpha- and beta-chain heterodimerization, which is required for receptor activation but not high-affinity binding“, *Mol Cell Biol*, Bd. 16, Nr. 6, S. 3035–3046, Juni 1996, doi: 10.1128/MCB.16.6.3035.

[44] J. M. Woodcock *u. a.*, „Three residues in the common beta chain of the human GM-CSF, IL-3 and IL-5 receptors are essential for GM-CSF and IL-5 but not IL-3 high affinity binding and interact with Glu21 of GM-CSF“, *EMBO J*, Bd. 13, Nr. 21, S. 5176–5185, Nov. 1994.

[45] S. Doulatov *u. a.*, „Induction of multipotential hematopoietic progenitors from human pluripotent stem cells via respecification of lineage-restricted precursors“, *Cell Stem Cell*, Bd. 13, Nr. 4, S. 459–470, Okt. 2013, doi: 10.1016/j.stem.2013.09.002.

[46] C. A. Hunter und S. A. Jones, „IL-6 as a keystone cytokine in health and disease“, *Nat Immunol*, Bd. 16, Nr. 5, S. 448–457, Mai 2015, doi: 10.1038/ni.3153.

[47] M. Murakami, D. Kamimura, und T. Hirano, „Pleiotropy and Specificity: Insights from the Interleukin 6 Family of Cytokines“, *Immunity*, Bd. 50, Nr. 4, S. 812–831, Apr. 2019, doi: 10.1016/j.immuni.2019.03.027.

[48] J. L. Zhao *u. a.*, „Conversion of danger signals into cytokine signals by hematopoietic stem and progenitor cells for regulation of stress-induced hematopoiesis“, *Cell Stem Cell*, Bd. 14, Nr. 4, S. 445–459, Apr. 2014, doi: 10.1016/j.stem.2014.01.007.

[49] K. Honda, A. Takaoka, und T. Taniguchi, „Type I interferon [corrected] gene induction by the interferon regulatory factor family of transcription factors“, *Immunity*, Bd. 25, Nr. 3, S. 349–360, Sep. 2006, doi: 10.1016/j.immuni.2006.08.009.

[50] D. Savitsky, T. Tamura, H. Yanai, und T. Taniguchi, „Regulation of immunity and oncogenesis by the IRF transcription factor family“, *Cancer Immunol Immunother*, Bd. 59, Nr. 4, S. 489–510, Apr. 2010, doi: 10.1007/s00262-009-0804-6.

[51] P. Lundberg *u. a.*, „Clonal evolution and clinical correlates of somatic mutations in myeloproliferative neoplasms“, *Blood*, Bd. 123, Nr. 14, S. 2220–2228, Apr. 2014, doi: 10.1182/blood-2013-11-537167.

[52] A. Elsässer *u. a.*, „The fusion protein AML1-ETO in acute myeloid leukemia with translocation t(8;21) induces c-jun protein expression via the proximal AP-1 site of the c-jun promoter in an indirect, JNK-dependent manner“, *Oncogene*, Bd. 22, Nr. 36, S. 5646–5657, Aug. 2003, doi: 10.1038/sj.onc.1206673.

[53] J. Rangatia *u. a.*, „Elevated c-Jun expression in acute myeloid leukemias inhibits C/EBPalpha DNA binding via leucine zipper domain interaction“, *Oncogene*, Bd. 22, Nr. 30, S. 4760–4764, Juli 2003, doi: 10.1038/sj.onc.1206664.

[54] P. K. Vogt, „Jun, the oncoprotein“, *Oncogene*, Bd. 20, Nr. 19, S. 2365–2377, Apr. 2001, doi: 10.1038/sj.onc.1204443.

[55] C. Zhou *u. a.*, „JUN is a key transcriptional regulator of the unfolded protein response in acute myeloid leukemia“, *Leukemia*, Bd. 31, Nr. 5, S. 1196–1205, Mai 2017, doi: 10.1038/leu.2016.329.

[56] J. L. Bos, „ras oncogenes in human cancer: a review“, *Cancer Res*, Bd. 49, Nr. 17, S. 4682–4689, Sep. 1989.

[57] J. Cicenas *u. a.*, „KRAS, NRAS and BRAF mutations in colorectal cancer and melanoma“, *Med Oncol*, Bd. 34, Nr. 2, S. 26, Feb. 2017, doi: 10.1007/s12032-016-0879-9.

[58] S. Lanfredini, A. Thapa, und E. O'Neill, „RAS in pancreatic cancer“, *Biochem Soc Trans*, Bd. 47, Nr. 4, S. 961–972, Aug. 2019, doi: 10.1042/BST20170521.

[59] X. Liu *u. a.*, „RAS mutations in acute myeloid leukaemia patients: A review and meta-analysis“, *Clin Chim Acta*, Bd. 489, S. 254–260, Feb. 2019, doi: 10.1016/j.cca.2018.08.040.

[60] Y. Li *u. a.*, „Amplification of LAPTM4B and YWHAZ contributes to chemotherapy resistance and recurrence of breast cancer“, *Nat Med*, Bd. 16, Nr. 2, S. 214–218, Feb. 2010, doi: 10.1038/nm.2090.

[61] A. Mosquera Orgueira *u. a.*, „Personalized Survival Prediction of Patients With Acute Myeloblastic Leukemia Using Gene Expression Profiling“, *Front Oncol*, Bd. 11, S. 657191, 2021, doi: 10.3389/fonc.2021.657191.

[62] S. Vergarajauregui, J. A. Martina, und R. Puertollano, „LAPTMs regulate lysosomal function and interact with mucolipin 1: new clues for understanding mucolipidosis type IV“, *J Cell Sci*, Bd. 124, Nr. Pt 3, S. 459–468, Feb. 2011, doi: 10.1242/jcs.076240.

[63] J. De Rijck, K. Bartholomeeusen, H. Ceulemans, Z. Debyser, und R. Gijsbers, „High-resolution profiling of the LEDGF/p75 chromatin interaction in the ENCODE region“, *Nucleic Acids Res*, Bd. 38, Nr. 18, S. 6135–6147, Okt. 2010, doi: 10.1093/nar/gkq410.

[64] J. O. Eidahl *u. a.*, „Structural basis for high-affinity binding of LEDGF PWWP to mononucleosomes“, *Nucleic Acids Res*, Bd. 41, Nr. 6, S. 3924–3936, Apr. 2013, doi: 10.1093/nar/gkt074.

[65] S. El Ashkar *u. a.*, „LEDGF/p75 is dispensable for hematopoiesis but essential for MLL-rearranged leukemogenesis“, *Blood*, Bd. 131, Nr. 1, S. 95–107, Jan. 2018, doi: 10.1182/blood-2017-05-786962.

[66] M. M. Pradeepa, H. G. Sutherland, J. Ule, G. R. Grimes, und W. A. Bickmore, „Psip1/Ledgf p52 binds methylated histone H3K36 and splicing factors and contributes to the regulation of alternative splicing“, *PLoS Genet*, Bd. 8, Nr. 5, S. e1002717, 2012, doi: 10.1371/journal.pgen.1002717.

[67] A. Yokoyama und M. L. Cleary, „Menin critically links MLL proteins with LEDGF on cancer-associated target genes“, *Cancer Cell*, Bd. 14, Nr. 1, S. 36–46, Juli 2008, doi: 10.1016/j.ccr.2008.05.003.

[68] C. Mosimann, G. Hausmann, und K. Basler, „Parafibromin/Hyrax activates Wnt/Wg target gene transcription by direct association with beta-catenin/Armadillo“, *Cell*, Bd. 125, Nr. 2, S. 327–341, Apr. 2006, doi: 10.1016/j.cell.2006.01.053.

[69] A. G. Muntean *u. a.*, „The PAF complex synergizes with MLL fusion proteins at HOX loci to promote leukemogenesis“, *Cancer Cell*, Bd. 17, Nr. 6, S. 609–621, Juni 2010, doi: 10.1016/j.ccr.2010.04.012.

[70] B. Zhu *u. a.*, „Monoubiquitination of human histone H2B: the factors involved and their roles in HOX gene regulation“, *Mol Cell*, Bd. 20, Nr. 4, S. 601–611, Nov. 2005, doi: 10.1016/j.molcel.2005.09.025.
[71] K. El Omari *u. a.*, „Structure of the leukemia oncogene LMO2: implications for the assembly of a hematopoietic transcription factor complex“, *Blood*, Bd. 117, Nr. 7, S. 2146–2156, Feb. 2011, doi: 10.1182/blood-2010-07-293357.
[72] M. Sang, L. Ma, M. Sang, X. Zhou, W. Gao, und C. Geng, „LIM-domain-only proteins: multifunctional nuclear transcription coregulators that interacts with diverse proteins“, *Mol Biol Rep*, Bd. 41, Nr. 2, S. 1067–1073, Feb. 2014, doi: 10.1007/s11033-013-2952-1.
[73] I. A. Wadman *u. a.*, „The LIM-only protein Lmo2 is a bridging molecule assembling an erythroid, DNA-binding complex which includes the TAL1, E47, GATA-1 and Ldb1/NLI proteins“, *EMBO J*, Bd. 16, Nr. 11, S. 3145–3157, Juni 1997, doi: 10.1093/emboj/16.11.3145.
[74] E. Metzger *u. a.*, „LSD1 demethylates repressive histone marks to promote androgen-receptor-dependent transcription“, *Nature*, Bd. 437, Nr. 7057, S. 436–439, Sep. 2005, doi: 10.1038/nature04020.
[75] Y. Shi *u. a.*, „Histone demethylation mediated by the nuclear amine oxidase homolog LSD1“, *Cell*, Bd. 119, Nr. 7, S. 941–953, Dez. 2004, doi: 10.1016/j.cell.2004.12.012.
[76] S. Zhang, M. Liu, Y. Yao, B. Yu, und H. Liu, „Targeting LSD1 for acute myeloid leukemia (AML) treatment“, *Pharmacol Res*, Bd. 164, S. 105335, Feb. 2021, doi: 10.1016/j.phrs.2020.105335.
[77] S. Fletcher, „MCL-1 inhibitors - where are we now (2019)?“, *Expert Opin Ther Pat*, Bd. 29, Nr. 11, S. 909–919, Nov. 2019, doi: 10.1080/13543776.2019.1672661.
[78] E. Eklund, „The role of Hox proteins in leukemogenesis: insights into key regulatory events in hematopoiesis“, *Crit Rev Oncog*, Bd. 16, Nr. 1–2, S. 65–76, 2011, doi: 10.1615/critrevoncog.v16.i1-2.70.
[79] Y. Okada *u. a.*, „Homeodomain proteins MEIS1 and PBXs regulate the lineage-specific transcription of the platelet factor 4 gene“, *Blood*, Bd. 101, Nr. 12, S. 4748–4756, Juni 2003, doi: 10.1182/blood-2002-02-0380.
[80] P. A. Del Rizzo und R. C. Trievel, „Substrate and product specificities of SET domain methyltransferases“, *Epigenetics*, Bd. 6, Nr. 9, S. 1059–1067, Sep. 2011, doi: 10.4161/epi.6.9.16069.
[81] D. G. Grier, A. Thompson, A. Kwasniewska, G. J. McGonigle, H. L. Halliday, und T. R. Lappin, „The pathophysiology of HOX genes and their role in cancer“, *J Pathol*, Bd. 205, Nr. 2, S. 154–171, Jan. 2005, doi: 10.1002/path.1710.
[82] R. Marschalek, „MLL leukemia and future treatment strategies“, *Arch Pharm (Weinheim)*, Bd. 348, Nr. 4, S. 221–228, Apr. 2015, doi: 10.1002/ardp.201400449.
[83] K. Deguchi *u. a.*, „MOZ-TIF2-induced acute myeloid leukemia requires the MOZ nucleosome binding motif and TIF2-mediated recruitment of CBP“, *Cancer Cell*, Bd. 3, Nr. 3, S. 259–271, März 2003, doi: 10.1016/s1535-6108(03)00051-5.
[84] I. Kitabayashi, Y. Aikawa, L. A. Nguyen, A. Yokoyama, und M. Ohki, „Activation of AML1-mediated transcription by MOZ and inhibition by the MOZ-CBP fusion protein“, *EMBO J*, Bd. 20, Nr. 24, S. 7184–7196, Dez. 2001, doi: 10.1093/emboj/20.24.7184.
[85] C. V. Dang, „MYC on the path to cancer“, *Cell*, Bd. 149, Nr. 1, S. 22–35, März 2012, doi: 10.1016/j.cell.2012.03.003.
[86] X. Dolcet, D. Llobet, J. Pallares, und X. Matias-Guiu, „NF-kB in development and progression of human cancer“, *Virchows Arch*, Bd. 446, Nr. 5, S. 475–482, Mai 2005, doi: 10.1007/s00428-005-1264-9.
[87] P. Pai und S. Sukumar, „HOX genes and the NF-κB pathway: A convergence of developmental biology, inflammation and cancer biology“, *Biochim Biophys Acta Rev Cancer*, Bd. 1874, Nr. 2, S. 188450, Dez. 2020, doi: 10.1016/j.bbcan.2020.188450.
[88] J. J. Gasiorek und V. Blank, „Regulation and function of the NFE2 transcription factor in hematopoietic and non-hematopoietic cells“, *Cell Mol Life Sci*, Bd. 72, Nr. 12, S. 2323–2335, Juni 2015, doi: 10.1007/s00018-015-1866-6.
[89] P. S. Goerttler *u. a.*, „Gene expression profiling in polycythaemia vera: overexpression of transcription factor NF-E2“, *Br J Haematol*, Bd. 129, Nr. 1, S. 138–150, Apr. 2005, doi: 10.1111/j.1365-2141.2005.05416.x.
[90] J. S. Jutzi *u. a.*, „MPN patients harbor recurrent truncating mutations in transcription factor NF-E2“, *J Exp Med*, Bd. 210, Nr. 5, S. 1003–1019, Mai 2013, doi: 10.1084/jem.20120521.
[91] K. B. Kaufmann *u. a.*, „A novel murine model of myeloproliferative disorders generated by overexpression of the transcription factor NF-E2“, *J Exp Med*, Bd. 209, Nr. 1, S. 35–50, Jan. 2012, doi: 10.1084/jem.20110540.
[92] W. Wang, S. Schwemmers, E. O. Hexner, und H. L. Pahl, „AML1 is overexpressed in patients with myeloproliferative neoplasms and mediates JAK2V617F-independent overexpression of NF-E2“, *Blood*, Bd. 116, Nr. 2, S. 254–266, Juli 2010, doi: 10.1182/blood-2009-11-254664.
[93] C. Wiesmann, M. H. Ultsch, S. H. Bass, und A. M. de Vos, „Crystal structure of nerve growth factor in complex with the ligand-binding domain of the TrkA receptor“, *Nature*, Bd. 401, Nr. 6749, S. 184–188, Sep. 1999, doi: 10.1038/43705.
[94] C. Wiesmann und A. M. de Vos, „Nerve growth factor: structure and function“, *Cell Mol Life Sci*, Bd. 58, Nr. 5–6, S. 748–759, Mai 2001, doi: 10.1007/pl00000898.
[95] A. C. Gingras, B. Raught, und N. Sonenberg, „eIF4 initiation factors: effectors of mRNA recruitment to ribosomes and regulators of translation“, *Annu Rev Biochem*, Bd. 68, S. 913–963, 1999, doi: 10.1146/annurev.biochem.68.1.913.
[96] H. M. Simmons, B. L. Ruis, M. Kapoor, A. W. Hudacek, und K. F. Conklin, „Identification of NOM1, a nucleolar, eIF4A binding protein encoded within the chromosome 7q36 breakpoint region targeted in cases of pediatric acute myeloid leukemia“, *Gene*, Bd. 347, Nr. 1, S. 137–145, Feb. 2005, doi: 10.1016/j.gene.2004.12.027.
[97] K. Barford, C. C. Yap, N. D. Dwyer, und B. Winckler, „The related neuronal endosomal proteins NEEP21 (Nsg1) and P19 (Nsg2) have divergent expression profiles in vivo“, *J Comp Neurol*, Bd. 525, Nr. 8, S. 1861–1878, Juni 2017, doi: 10.1002/cne.24168.
[98] P. Chander, M. J. Kennedy, B. Winckler, und J. P. Weick, „Neuron-Specific Gene 2 (NSG2) Encodes an AMPA Receptor Interacting Protein That Modulates Excitatory Neurotransmission“, *eNeuro*, Bd. 6, Nr. 1, S. ENEURO.0292-18.2018, Feb. 2019, doi: 10.1523/ENEURO.0292-18.2018.
[99] N. Muthusamy, Y.-J. Chen, D.-M. Yin, L. Mei, und C. Bergson, „Complementary roles of the neuron-enriched endosomal proteins NEEP21 and calcyon in neuronal vesicle trafficking“, *J Neurochem*, Bd. 132, Nr. 1, S. 20–31, Jan. 2015, doi: 10.1111/jnc.12989.
[100] T. Ueda *u. a.*, „Fbxl10 overexpression in murine hematopoietic stem cells induces leukemia involving metabolic activation and upregulation of Nsg2“, *Blood*, Bd. 125, Nr. 22, S. 3437–3446, Mai 2015, doi: 10.1182/blood-2014-03-562694.
[101] Y. Arai *u. a.*, „The inv(11)(p15q22) chromosome translocation of de novo and therapy-related myeloid malignancies results in fusion of the nucleoporin gene, NUP98, with the putative RNA helicase gene, DDX10“, *Blood*, Bd. 89, Nr. 11, S. 3936–3944, Juni 1997.
[102] M. Iwamoto, H. Asakawa, Y. Hiraoka, und T. Haraguchi, „Nucleoporin Nup98: a gatekeeper in the eukaryotic kingdoms“, *Genes Cells*, Bd. 15, Nr. 7, S. 661–669, Juni 2010, doi: 10.1111/j.1365-2443.2010.01415.x.
[103] N. L. Michmerhuizen, J. M. Klco, und C. G. Mullighan, „Mechanistic insights and potential therapeutic approaches for NUP98-rearranged hematologic malignancies“, *Blood*, Bd. 136, Nr. 20, S. 2275–2289, Nov. 2020, doi: 10.1182/blood.2020007093.

[104] G. J. Hannon und D. Beach, „p15INK4B is a potential effector of TGF-beta-induced cell cycle arrest“, *Nature*, Bd. 371, Nr. 6494, S. 257–261, Sep. 1994, doi: 10.1038/371257a0.
[105] Y. Kotake, M. Naemura, C. Murasaki, Y. Inoue, und H. Okamoto, „Transcriptional Regulation of the p16 Tumor Suppressor Gene“, *Anticancer Res*, Bd. 35, Nr. 8, S. 4397–4401, Aug. 2015.
[106] W. Yu *u. a.*, „Epigenetic silencing of tumour suppressor gene p15 by its antisense RNA“, *Nature*, Bd. 451, Nr. 7175, S. 202–206, Jan. 2008, doi: 10.1038/nature06468.
[107] K. Chaudhary, S. Deb, N. Moniaux, M. P. Ponnusamy, und S. K. Batra, „Human RNA polymerase II-associated factor complex: dysregulation in cancer“, *Oncogene*, Bd. 26, Nr. 54, S. 7499–7507, Nov. 2007, doi: 10.1038/sj.onc.1210582.
[108] J. Serio *u. a.*, „The PAF complex regulation of Prmt5 facilitates the progression and maintenance of MLL fusion leukemia“, *Oncogene*, Bd. 37, Nr. 4, S. 450–460, Jan. 2018, doi: 10.1038/onc.2017.337.
[109] R. Bernardi und P. P. Pandolfi, „Structure, dynamics and functions of promyelocytic leukaemia nuclear bodies“, *Nat Rev Mol Cell Biol*, Bd. 8, Nr. 12, S. 1006–1016, Dez. 2007, doi: 10.1038/nrm2277.
[110] H. de Thé, P. P. Pandolfi, und Z. Chen, „Acute Promyelocytic Leukemia: A Paradigm for Oncoprotein-Targeted Cure“, *Cancer Cell*, Bd. 32, Nr. 5, S. 552–560, Nov. 2017, doi: 10.1016/j.ccell.2017.10.002.
[111] F. Grignani *u. a.*, „Effects on differentiation by the promyelocytic leukemia PML/RARalpha protein depend on the fusion of the PML protein dimerization and RARalpha DNA binding domains“, *EMBO J*, Bd. 15, Nr. 18, S. 4949–4958, Sep. 1996.
[112] U. Sahin, V. Lallemand-Breitenbach, und H. de Thé, „PML nuclear bodies: regulation, function and therapeutic perspectives“, *J Pathol*, Bd. 234, Nr. 3, S. 289–291, Nov. 2014, doi: 10.1002/path.4426.
[113] L. Duciel, L. C. Monraz Gomez, M. Kondratova, I. Kuperstein, und S. Saule, „The Phosphatase PRL-3 Is Involved in Key Steps of Cancer Metastasis“, *J Mol Biol*, Bd. 431, Nr. 17, S. 3056–3067, Aug. 2019, doi: 10.1016/j.jmb.2019.06.008.
[114] J. Zhou *u. a.*, „PRL-3, a metastasis associated tyrosine phosphatase, is involved in FLT3-ITD signaling and implicated in anti-AML therapy“, *PLoS One*, Bd. 6, Nr. 5, S. e19798, Mai 2011, doi: 10.1371/journal.pone.0019798.
[115] null Siddikuzzaman, C. Guruvayoorappan, und V. M. Berlin Grace, „All trans retinoic acid and cancer“, *Immunopharmacol Immunotoxicol*, Bd. 33, Nr. 2, S. 241–249, Juni 2011, doi: 10.3109/08923973.2010.521507.
[116] M. Ichikawa, A. Yoshimi, M. Nakagawa, N. Nishimoto, N. Watanabe-Okochi, und M. Kurokawa, „A role for RUNX1 in hematopoiesis and myeloid leukemia“, *Int J Hematol*, Bd. 97, Nr. 6, S. 726–734, Juni 2013, doi: 10.1007/s12185-013-1347-3.
[117] L. E. Swart und O. Heidenreich, „The RUNX1/RUNX1T1 network: translating insights into therapeutic options“, *Exp Hematol*, Bd. 94, S. 1–10, Feb. 2021, doi: 10.1016/j.exphem.2020.11.005.
[118] J. N. Davis, L. McGhee, und S. Meyers, „The ETO (MTG8) gene family“, *Gene*, Bd. 303, S. 1–10, Jan. 2003, doi: 10.1016/s0378-1119(02)01172-1.
[119] A. M. Melnick *u. a.*, „The ETO protein disrupted in t(8;21)-associated acute myeloid leukemia is a corepressor for the promyelocytic leukemia zinc finger protein“, *Mol Cell Biol*, Bd. 20, Nr. 6, S. 2075–2086, März 2000, doi: 10.1128/MCB.20.6.2075-2086.2000.
[120] S. Rossetti, A. T. Hoogeveen, und N. Sacchi, „The MTG proteins: chromatin repression players with a passion for networking“, *Genomics*, Bd. 84, Nr. 1, S. 1–9, Juli 2004, doi: 10.1016/j.ygeno.2004.02.011.
[121] X.-J. Sun *u. a.*, „A stable transcription factor complex nucleated by oligomeric AML1-ETO controls leukaemogenesis“, *Nature*, Bd. 500, Nr. 7460, S. 93–97, Aug. 2013, doi: 10.1038/nature12287.
[122] M. I. Hood und E. P. Skaar, „Nutritional immunity: transition metals at the pathogen-host interface“, *Nat Rev Microbiol*, Bd. 10, Nr. 8, S. 525–537, Juli 2012, doi: 10.1038/nrmicro2836.
[123] M. Laouedj *u. a.*, „S100A9 induces differentiation of acute myeloid leukemia cells through TLR4“, *Blood*, Bd. 129, Nr. 14, S. 1980–1990, Apr. 2017, doi: 10.1182/blood-2016-09-738005.
[124] I. Stríz und I. Trebichavský, „Calprotectin - a pleiotropic molecule in acute and chronic inflammation“, *Physiol Res*, Bd. 53, Nr. 3, S. 245–253, 2004.
[125] P. F. van Rheenen, E. Van de Vijver, und V. Fidler, „Faecal calprotectin for screening of patients with suspected inflammatory bowel disease: diagnostic meta-analysis“, *BMJ*, Bd. 341, S. c3369, Juli 2010, doi: 10.1136/bmj.c3369.
[126] P. D. Aplan, K. Nakahara, S. H. Orkin, und I. R. Kirsch, „The SCL gene product: a positive regulator of erythroid differentiation“, *EMBO J*, Bd. 11, Nr. 11, S. 4073–4081, Nov. 1992.
[127] T. Hoang, J. A. Lambert, und R. Martin, „SCL/TAL1 in Hematopoiesis and Cellular Reprogramming“, *Curr Top Dev Biol*, Bd. 118, S. 163–204, 2016, doi: 10.1016/bs.ctdb.2016.01.004.
[128] L. Robb und C. G. Begley, „The SCL/TAL1 gene: roles in normal and malignant haematopoiesis“, *Bioessays*, Bd. 19, Nr. 7, S. 607–613, Juli 1997, doi: 10.1002/bies.950190711.
[129] M. R. Imperato, P. Cauchy, N. Obier, und C. Bonifer, „The RUNX1-PU.1 axis in the control of hematopoiesis“, *Int J Hematol*, Bd. 101, Nr. 4, S. 319–329, Apr. 2015, doi: 10.1007/s12185-015-1762-8.
[130] P. Kastner und S. Chan, „PU.1: a crucial and versatile player in hematopoiesis and leukemia“, *Int J Biochem Cell Biol*, Bd. 40, Nr. 1, S. 22–27, 2008, doi: 10.1016/j.biocel.2007.01.026.
[131] M. J. Klemsz, S. R. McKercher, A. Celada, C. Van Beveren, und R. A. Maki, „The macrophage and B cell-specific transcription factor PU.1 is related to the ets oncogene“, *Cell*, Bd. 61, Nr. 1, S. 113–124, Apr. 1990, doi: 10.1016/0092-8674(90)90219-5.
[132] B. Groner und V. von Manstein, „Jak Stat signaling and cancer: Opportunities, benefits and side effects of targeted inhibition“, *Mol Cell Endocrinol*, Bd. 451, S. 1–14, Aug. 2017, doi: 10.1016/j.mce.2017.05.033.
[133] G. R. Stark und J. E. Darnell, „The JAK-STAT pathway at twenty“, *Immunity*, Bd. 36, Nr. 4, S. 503–514, Apr. 2012, doi: 10.1016/j.immuni.2012.03.013.
[134] J. K. Friedrich, K. I. Panov, P. Cabart, J. Russell, und J. C. B. M. Zomerdijk, „TBP-TAF complex SL1 directs RNA polymerase I pre-initiation complex formation and stabilizes upstream binding factor at the rDNA promoter“, *J Biol Chem*, Bd. 280, Nr. 33, S. 29551–29558, Aug. 2005, doi: 10.1074/jbc.M501595200.
[135] J. Heix, J. C. Zomerdijk, A. Ravanpay, R. Tjian, und I. Grummt, „Cloning of murine RNA polymerase I-specific TAF factors: conserved interactions between the subunits of the species-specific transcription initiation factor TIF-IB/SL1“, *Proc Natl Acad Sci U S A*, Bd. 94, Nr. 5, S. 1733–1738, März 1997, doi: 10.1073/pnas.94.5.1733.
[136] S. Naidu, J. K. Friedrich, J. Russell, und J. C. B. M. Zomerdijk, „TAF1B is a TFIIB-like component of the basal transcription machinery for RNA polymerase I“, *Science*, Bd. 333, Nr. 6049, S. 1640–1642, Sep. 2011, doi: 10.1126/science.1207656.
[137] B. Stoleru *u. a.*, „Tropomyosin-receptor-kinases signaling in the nervous system“, *Maedica (Bucur)*, Bd. 8, Nr. 1, S. 43–48, März 2013.
